# Hour-Glass Stomach

**Published:** 1914-08

**Authors:** 


**Hour-Glass Stomach.** Holland, Liverpool Med.-Chir. Jour., has had 34 cases, 32 in women. Only two had been diagnosed positively and only 10 tentatively, prior to X-ray examination.

